# Computer-Aided Grading of Gliomas Combining Automatic Segmentation and Radiomics

**DOI:** 10.1155/2018/2512037

**Published:** 2018-05-08

**Authors:** Wei Chen, Boqiang Liu, Suting Peng, Jiawei Sun, Xu Qiao

**Affiliations:** Department of Biomedical Engineering, School of Control Science and Engineering, Shandong University, Shandong, China

## Abstract

Gliomas are the most common primary brain tumors, and the objective grading is of great importance for treatment. This paper presents an automatic computer-aided diagnosis of gliomas that combines automatic segmentation and radiomics, which can improve the diagnostic ability. The MRI data containing 220 high-grade gliomas and 54 low-grade gliomas are used to evaluate our system. A multiscale 3D convolutional neural network is trained to segment whole tumor regions. A wide range of radiomic features including first-order features, shape features, and texture features is extracted. By using support vector machines with recursive feature elimination for feature selection, a CAD system that has an extreme gradient boosting classifier with a 5-fold cross-validation is constructed for the grading of gliomas. Our CAD system is highly effective for the grading of gliomas with an accuracy of 91.27%, a weighted macroprecision of 91.27%, a weighted macrorecall of 91.27%, and a weighted macro-*F*1 score of 90.64%. This demonstrates that the proposed CAD system can assist radiologists for high accurate grading of gliomas and has the potential for clinical applications.

## 1. Introduction

Gliomas are the most common primary brain tumors, characterized by the uncontrolled proliferation of abnormal brain cells. This disease is one of the most common causes of cancer death in men and women [1]. According to classification of the World Health Organization (WHO), gliomas can be subdivided by their malignancy into grade II (lower grade) to grade IV (high grade) [2]. The clinical patients with high-grade gliomas, such as glioblastomas, have the median overall survival rate of 23.1 months, the 2-year survival rate of 47.4%, and the 4-year survival rate of 18.5% [3]. On the contrast, the slower growing low-grade gliomas, such as astrocytomas and oligodendrogliomas, come with an overall 10-year survival rate of 57% [4]. Therefore, early detection is considered as an effective way to get a hopeful prognosis.

Modern imaging techniques allow clinicians and radiologists to evaluate the progression of tumors and choose optimal treatment strategy, without invasive neurosurgery. There are many imaging modalities that can be used to study the brain, such as computed tomography (CT), positron emission tomography (PET), and magnetic resonance imaging (MRI). These imaging modalities can provide effective and reliable information about brain tissues. In view of the advantages of high soft tissues contrast and high spatial resolution, MRI is widely used to evaluate the tumor heterogeneity [5]. However, there are several common MRI modalities including T1-weighted (T1), T2-weighted (T2), gadolinium enhanced T1-weighted (T1c), and Fluid-Attenuated Inversion Recovery (FLAIR), which generate a large number of medical images. This has become a huge burden for radiologists, resulting in inaccurate detection or misinterpretation. Therefore, with the development of computer technology, there is an increasing demand for computer-aided diagnosis.

Recently, computer-aided detection diagnosis (CAD) has gradually become a research hotspot in the area of medical imaging. The main idea of CAD is to assist radiologists in making clinical decision by using the report of computer system as a “second opinion” [6]. El-Dahshan et al. [6] developed a CAD system that used feedback pulse-coupled neural network for image segmentation, discrete wavelet transform for features extraction, the principal component analysis for feature reducing, and the feed forward backpropagation neural network for classifying inputs into normal or abnormal. Zacharaki et al. [7] suggested that MRI texture and shape features in a machine learning scheme can help to evaluate the malignancy of brain tumor and identify certain tumor. The CAD system based on the use of automatic segmentation and ensemble classification techniques can accurately classify brain tumor as benign or malignant [8]. All of these studies have shown that CAD can help to improve the accuracy and efficiency of the diagnostic process and reduce the burden of work.

In the meantime, a technology called radiomics has developed rapidly, which explores the correlation between medical images and underlying genetic characteristics using a large number of automated data characterization algorithms to transform the region of interest (ROI) into high-throughput quantitative features. In 2008, Diehn et al. [9] found that the image features and genetic characteristics are highly correlated through the study of glioblastoma multiforme. The degree of enhancement within the tumor can indirectly reflect the expression level of epithelial growth factor receptor (EGFR). Patients with an infiltrative imaging phenotype have more tendency to have multiple tumor foci and show significantly shorter survival than the corresponding counterparts. In 2012, Dutch scholar Lambin et al. [10] formally proposed the concept of radiomics; that is, high-throughput extraction of a large number of image features from radiological images and the adoption of a large number of automated data characterization algorithms transform the image data in the ROI to high-resolution and exploitable spatial data. Hsieh et al. [11] proposed a computer-aided diagnosis system based on local and global MRI features to predict the malignancy of diffuse gliomas. The proposed image features have great potential in distinguishing glioblastomas from lower-grade gliomas. Lao et al. [12] proposed a deep learning-based radiomics model that has better performance than conventional models relying on explicitly designed handcrafted features for survival prediction in glioblastoma multiforme.

However, many CAD systems ignore the meaning of the features in the process of classification. Meanwhile, most radiomics studies focus on manual segmentation based ROI, which largely limit the development of radiomics. In this paper, we present an automatic computer-aided diagnosis for gliomas grading that combines automatic segmentation and radiomics. We use the MRI data provided by MICCAI Brain Tumor Segmentation Challenge 2015 containing 220 high-grade gliomas and 54 low-grade gliomas to evaluate our system. A multiscale 3D convolutional neural network is trained to segment whole tumor regions. Compared with the ground truth, satisfying segmentations of the whole tumor regions are obtained. A wide range of radiomic features including first-order features, shape features, and texture features is extracted from segmented tumor regions. Using support vector machines with recursive feature elimination for feature selection, we construct a 20-feature radiomic signature and train an extreme gradient boosting classifier. Robustness and accuracy results are obtained via 5-fold cross-validation. The experimental results show that the proposed CAD system is highly effective in the grading of gliomas. We further analyze the selected feature subset for gliomas grading. We also test our system using ground truth segmentation and achieve comparable results. The system is automated and general and, if a significant number of subjects exist, has the potential for clinical applications.

## 2. Method

In this section, the methods involved in the CAD system are mainly introduced, which are organized as 5 parts: data preprocessing, automated tumor segmentation, radiomic features extraction, feature selection, and data classification.

### 2.1. Preprocessing

One difficulty in dealing with MRI scans is to deal with the artifacts caused by the inhomogeneity of the magnetic field and the small motions that the patient produces during scanning. The existence of artifacts has a great impact on the segmentation performance. N4ITK [13] is a widely used algorithm for removing bias field. In addition, the intensities may vary significantly between different scans because MRI scans were acquired from multiple institutions. Therefore, histogram matching algorithm [14] is used to transform each image to a specified histogram to ensure that all the images have the similar gray level ranges.

### 2.2. Segmentation

Segmentation is the most important and challenging part of a CAD system. The purpose of segmentation is to extract the ROI that is of great value to the diagnosis. This is a challenging task because of the unclear boundary of gliomas. Furthermore, gliomas may appear anywhere in the brain with different shapes and sizes. A lot of automated methods have been developed for brain tumor segmentation problem, including generative modeling method and discriminative model method. The former method relies on the prior knowledge of health and oncology tissue and the latter method relies on a large number of features extracted from the raw image such as local histograms and texture features. Relying on traditional machine learning methods, these segmentation methods have achieved great success. However, these methods may not be able to take full advantage of the training data due to the complexity of medical images. Recently, deep learning methods, especially convolutional neural networks (CNN), have led to great success in the field of medical image processing, such as classification and segmentation of medical images. These approaches incorporate feature engineering steps into learning steps that allow learning a wider range of features, thus contributing to a more robust representation of image data.

The CNN-based methods are performed by stacking several convolutional layers to form a hierarchy of features. The stack of input planes is fed into the convolutional layers, producing an output containing several feature maps by the application of kernels. The kernels, often interchangeably called filters, are arrays of values representing some sort of feature.

**Figure 1:**
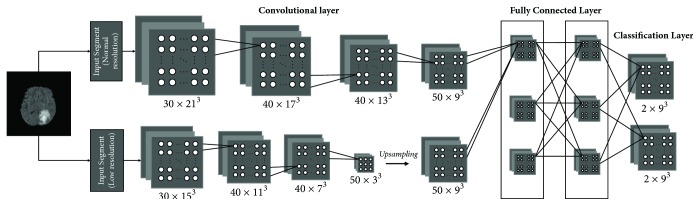
The basic architecture of DeepMedic.

The automatic brain tumor segmentation method we used for CAD is DeepMedic, which is a 11-layer deep, multiscale 3D CNN first presented by Kamnitsas et al. [15] for brain lesion segmentation.  Figure 1 is a simplified version of DeepMedic. As can be seen from the figure, the network consists of two pathways, one with normal resolution input segments and the remaining one with the lower resolution input segments. These segments then pass through a series of convolutional layers and two fully connected layers and finally a classification layer to produce soft segmentation maps.

One of the most prominent features of DeepMedic is the use of 3D CNNs as a basis for its architecture, which are characterized by convolving each layer's channels with a 3-dimensional kernel rather than traditional 2-dimensional kernel. This can be thought as a rectangular prism traversing the volume of the image at hand. Using these 3D CNNs can better represent volumetric data but will lead to increased computational costs. In order to overcome this problem, the dense inference technique was adopted. When the input image patch is larger than the CNN's receptive field, the classification layer can output multiple predictions.

In general, the deeper network with more convolutional layers has better discrimination power. This is attributed to the richer structures captured by the deeper models. However, with the deepening of the model, the computational cost is also getting higher and higher. To solve this problem, the small 3^3^ kernels with less weights are adopted. These smaller kernels have the advantage of faster convolving speed and fewer parameters, thus contributing to a significant decrease in computational cost.

Deeper networks are also harder to train. As the network becomes deeper and deeper, preservation of loss functions becomes more and more difficult due to the multiplication of the variance of the loss function as it propagates through each layer. In order to alleviate this obstacle, DeepMedic initializes the kernel weights with the normal distribution $𝒩\left(0,\sqrt{2/n_{l}^{in}}\right)$ where *n*_*l*_^in^ is the number of weights through which a neuron of layer *l* is connected to its input. Batch normalization is also used to deal with the issue of covariate shift.

### 2.3. Radiomic Features Extraction

From the perspective of brain tumor medical characteristics and clinical cognition, doctors mainly determine the tumor grade from a lot of points. The brain tumor intensities of MRI scans with different modalities may have high signal, low signal, or equal signal due to different internal components. According to the degree of intratumoral vessels, the amplitude of the signal enhancement after MRI enhanced scan is either high or low. The size and margins of the tumor may reflect the growth pattern of the tumor. Most brain tumors with uniform signal intensity are benign. In contrast, most brain tumors with nonuniform signals intensity are malignant. Higher-grade tumors usually show irregular shapes with larger size than lower-grade tumors. The high-grade tumors are more likely to appear to be significant enhancement of the signals, suggesting the destruction of the blood brain barrier, while low level tumors generally have no enhanced signals. The morphological and marginal features of the tumor can also provide some information to distinguish between benign and malignant tumors.

As mentioned above, conventional magnetic resonance imaging has been able to provide diagnostic information related to tumor grade, such as the location, shape, size, signal characteristics, mass effect, and peritumoral edema of brain tumors. However, these characteristics are often described subjectively and qualitatively. Recent advances in image analysis and machine learning facilitate the establishment of objective and accurate quantitative imaging descriptors that may be used as prognostic biomarkers [16].

Consistent with the features described by Imaging Biomarker Standardization Initiative (IBSI) [17], a wide range of radiomic features including first-order features, shape features, and texture features was extracted from the segmented brain tumor regions. First-order features (energy, entropy, standard deviation, skewness, kurtosis, etc.) describe the characteristics about voxel intensities and are calculated according to the intensity distribution and histogram. Shape features describe the three-dimensional size and shape of the tumors. These features can provide important diagnostic information; for example, the extent of tumor diffusion is usually measured by the longest diameter. Texture analysis contains a series of techniques that can quantify the gray-level pattern and the relationship of pixels through a wide range of statistical measures. Several studies have demonstrated the importance of texture features in evaluating the malignancy of gliomas [7, 11]. An extensive discussion of texture features can be seen in [18]. Inspired by them, we extracted a wider range of texture features as shown in Table 1. The detailed description of these features can be found in [17].

### 2.4. Feature Selection

Too many features will increase the computational cost, and the redundancy between features will reduce the accuracy of the classification. Furthermore, the number of features is more than the number of samples in this work, which will increase the probability of overfitting. Therefore, feature selection is essential. There are two main types of feature selection algorithms: filtering methods and wrapping methods. Filter methods use a statistical measure based on the inherent information of samples to compute a score for each feature. The features are then ranked according to their scores, and the top-ranked features are kept as the final features for classification. However, the selected features may contain many redundant features because the scores are calculated independently for each feature, completely ignoring the dependencies on other features. On the contrary, the wrapping methods consider a subset of features that have best discrimination power by evaluating the performance of the classifier using this feature subset. Therefore, wrapping-based feature selection methods is more suitable for selecting radiomic features because of their obvious correlation.

A quintessential example of wrapping methods is Support Vector Machine Recursive Feature Elimination (SVM-RFE) algorithm, which was proposed by Guyon et al. [19] for selecting gene data for cancer classification. SVM-RFE is a feature selection algorithm utilizing support vector machine (SVM) methods based on recursive feature elimination (RFE). SVM is a widely used classification technique based on the idea of finding a separating hyperplane that best divides a dataset into two classes [20]. The RFE method selects features by recursively considering smaller and smaller sets of features according to their weights assigned by an external estimator. To be specific, given training examples **X**_0_ = [**x**_1_, **x**_2_,…, **x**_*k*_,…, **x**_*l*_]^*T*^ and class labels *y* = [*y*_1_, *y*_2_,…,*y*_*k*_,…,*y*_*l*_]^*T*^; let **s** = [1,2,…, *n*] be the subset of surviving features. In each loop, an SVM classifier is trained on **s**, and the corresponding weight vector **w** is calculated. The features are then ranked based on **w**, and the features with smallest ranking criterion are removed from **s**. This procedure is repeated until **s** = [ ]. Thus, we can obtain the feature ranked list **r** using feature removing sequence, as the features removed later are more important. The features removed in the last iteration have the highest rankings. In order to find the optimal number of features to be selected, cross-validation can be used. The SVM-RFE with cross-validation starts with all the features, computes the cross-validated performance score, and removes the lowest *k* features. These procedures are repeated until all the features are eliminated. Finally, a feature subset with the best performance score can be obtained.

### 2.5. Classification

In order to solve the problem of a relatively small dataset in our work, an ensemble learning model is used to trade off the approximation error and estimation error. Ensemble learning model incorporates a variety of machine learning algorithms and typically achieves significantly superior generalization performance over a single learner. Gradient boosting tree (XGBoost) [21] is a kind of ensemble learning models for regression and classification problems, which generates a prediction model in the form of an ensemble of decision trees.

Given a dataset *𝒟* = {(**x**_*i*_, *y*_*i*_)}, the tree ensemble model can be seen as an addictive model consisting of *K* trees.

(1) $$\begin{array}{c} {\hat{y}}_{i}=\phi \left(\;x_{i}\;\right)=\overset{K}{\underset{k=1}{\sum }}f_{k}\left(\;x_{i}\;\right),\;f_{k}\in F, \end{array}$$

where *ℱ* is the tree space containing all possible regression trees. The function *f*_*k*_, which we need to learn, corresponds to the tree structure and leaf weights. In contrast to traditional decision tree, every leaf in the tree consists of a continuous score, and the final prediction is based on the summation of these scores. To learn the model, the following regularized objective will be minimized.

(2) Lϕ=∑inlyi,y^i+∑k=1KΩfkwhere  Ωf=γT+12λw2.

In (2), *l* is restricted to be a differentiable convex loss function that measures the difference between the prediction ${\hat{y}}_{i}$ and the target *y*_*i*_. The second term *Ω*(*f*) penalizes the complexity of tree in terms of the number of leaves in the tree *T* with coefficient *γ* and the vector of scores on leaves *w* with coefficient *λ*. Two additional techniques, shrinkage and column subsampling, are further used to reduce overfitting. Shrinkage reduces the influence of each tree by scaling newly added weights and thus leaves space for the following trees to improve the model while reducing the possibility of overfitting. Similar to random forest, column subsampling only considers the attributes of a random subset to establish a tree. This will not only improve the speed of training, but also help to reduce the overfitting.

## 3. Result

### 3.1. MRI Data

**Figure 2:**
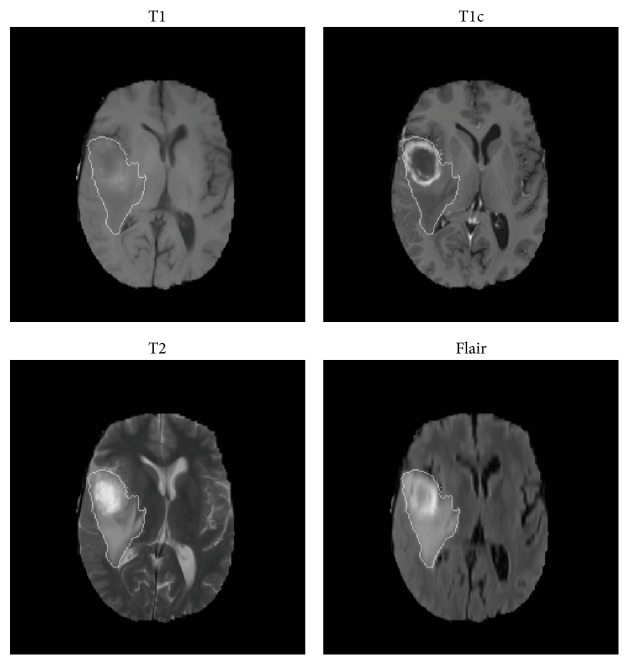
The whole tumor region with ground truth segmentation.

Experiments were carried out on the MR images obtained from BraTS'15 challenge [22], which contains 220 high-grade gliomas (HGG) and 54 low-grade gliomas (LGG). Each subject contains four modalities: native T1-weighted (T1), postcontrast T1-weighted (T1c), T2-weighted (T2), and T2 Fluid Attenuated Inversion Recovery (FLAIR). All subjects were confirmed pathologically by experts. The HGG group consists of anaplastic astrocytomas and glioblastoma multiforme tumors, and the LGG group consists of astrocytomas and oligoastrocytomas. All images were skull stripped and registered to the T1c image and resampled to 1 mm isotropic resolution within a standard axial orientation. All images were labeled into five parts: necrosis, edema, active-enhanced tumor, nonenhanced tumor, and normal region. In this work, we merged all tumor subregions into the whole tumor region as shown in Figure 2.

### 3.2. Preprocessing

**Figure 3:**
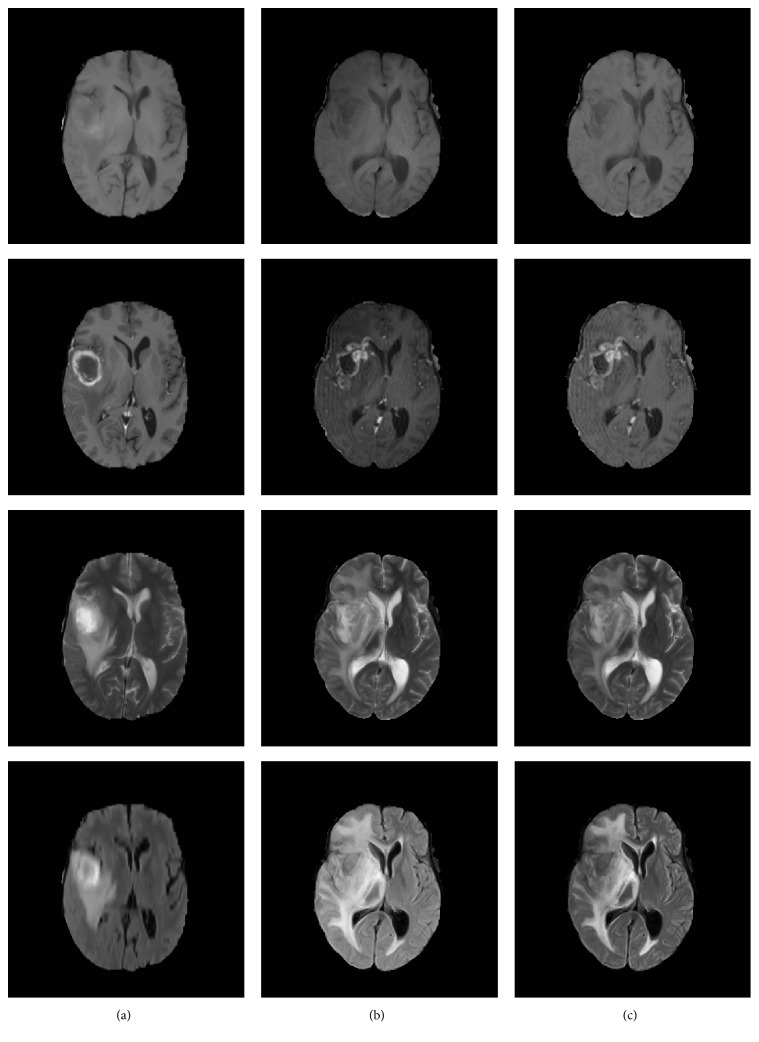
The results of preprocessing.

The N4ITK bias correction method was applied to all scans using advanced normalization tools (ANTS) [23]. Then the histogram matching algorithm was applied to transform each scan to a specified histogram using SimpleITK [24]. The images of “brats_2013_pat0001_1” were selected as reference samples. Then all images were normalized to the zero-mean and unit variance through subtracting the mean and being divided by the standard deviation. Some preprocessing results are shown in Figure 3. Column (a) shows the reference images, column (b) shows the images before preprocessing of a random selected subject, and column (c) shows the results after preprocessing.

### 3.3. Segmentation

The detailed architecture of the network is shown in Table 2. We have four modalities, that is, T1, T1c, T2, and FLAIR. Therefore, a total of four channels are fed into the network. The predicted labels are classified into two sets: whole tumor (including necrosis, edema, active-enhanced tumor, and nonenhanced tumor) and nontumor region. There are two parallel identical pathways, and the number of feature maps of each convolutional layer is [30,30,40,40,40,40,50,50]. The input dimensions of the normal resolution path is 25*∗*25*∗*25, and the input dimension of the low resolution path is 19*∗*19*∗*19. Two hidden layers with 150 feature maps follow the concatenation of the pathways. The network was trained on a GeForce GTX 1080 Ti GPU with a batch size of 10. RMSProp was used as the optimizer. The start learning rate was set to 0.001.

Multiple criteria were computed as performance metrics to quantify the segmentation results. Dice coefficient (see (3)) is the most commonly used metric for evaluating medical image segmentations [25]. *P* is the area that is predicted to be tumor and *G* is true tumor area. It measures the overlap between the automatic segmentations and ground truth with a value between 0 and 1. The higher the Dice score, the better the segmentation performance.

(3) $$\begin{array}{c} Dice\left(\;P,G\;\right)=\frac{P\cap G}{\left(\left|P\right|+\left|G\right|\right)/2}. \end{array}$$

Sensitivity and specificity are also commonly used statistical measures. The sensitivity, also called true positive rate, is defined as the proportion of positives that are correctly predicted. It measures the portion of tumor regions in the ground truth that are also predicted as tumor regions by the automatic segmentation method. The specificity, also called true negative rate, is defined as the proportion of negatives that are correctly predicted. It measures the portion of normal tissue regions in the ground truth that are also predicted as normal tissue regions by the automatic segmentation method.

**Figure 4:**
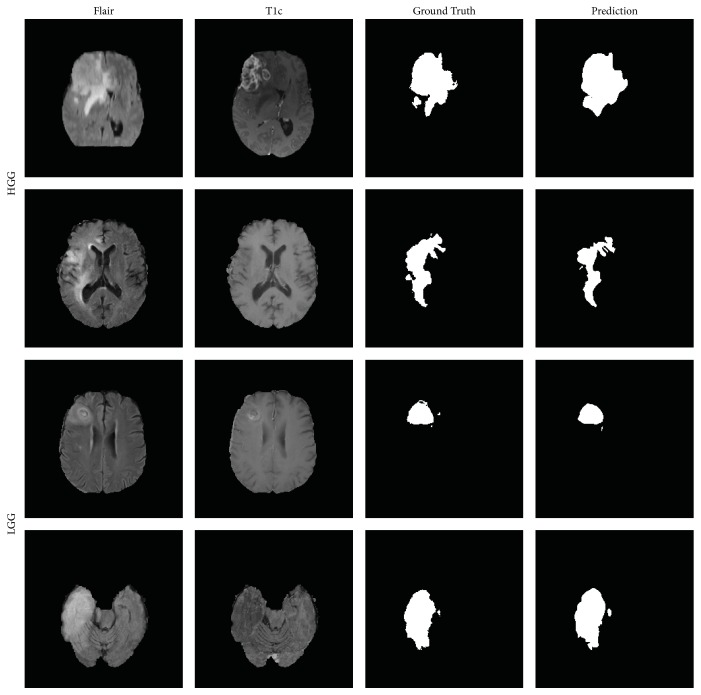
Examples of segmentation results.

We trained and evaluated our network on all the 274 subjects via 5-fold cross-validation and achieved a Dice score of 0.89, a sensitivity of 0.89, and a specificity of 0.90. Four examples of the automatic segmentations on testing data are shown in Figure 4. The first two rows depict the segmentations from HGG subjects, and the next two rows depict the segmentations from LGG subjects. Compared with the ground truth, satisfying segmentations of the whole tumor regions are obtained.

### 3.4. Radiomic Features Extraction and Selection

A wide range of radiomic features was extracted from the whole tumor regions. This step was performed by Pyradiomics [26], which can extract a wide range of radiomic features from brain tumor MRI. For each modality, 105 3D-Radiomic features were extracted, including 18 first-order features, 13 shape features, and 74 texture features. Thus, a total of 420 radiomic features were extracted from each subject.

Because of too many features, a feature selection step should be required prior to establishing a classification model. This step not only reduces overfitting but also improves generalization of the classification model. In order to select the most discriminating feature subset, we used 5-fold cross-validation. For each fold, SVM-RFE with leave-one-out cross-validation algorithm was used to find the optimal features that best predict the malignancy of gliomas. Finally, 20 features were commonly selected for all folds, as shown in Table 3. A detailed description of these features can be found in Pyradiomics documentation website (http://pyradiomics.readthedocs.io).

As we can see from Table 3, 1 feature from the T1 image, 12 features from the T1c image, 4 features from the T2 image, and 3 features from the FLAIR image were selected. The features from T1 image make up the majority, suggesting that the T1c image best reflects the difference between HGG and LGG. In terms of feature groups, none of the shape features were selected. These results are consistent with the research in [27].

### 3.5. Classification

Implementation of XGBoost was performed in python using the open-source code provided in [21]. In addition to XGBoost, two other pattern classification methods were investigated for comparison: Extremely Randomized Trees (ERT) [28] and support vector machine (SVM) [20]. Extremely Randomized Trees are similar to random forest but differ in two ways. Extremely Randomized Trees do not use the bagging procedure when building a tree. Like random forest, a random set of attributes were selected at every node. Extremely Randomized Trees go one step further and select the splitting attribute randomly, rather than finding the best split among random subset of variables. A SVM is a discriminative classifier based on the idea of finding a separating hyperplane that best divides a dataset into two classes. SVM is widely used for different tasks due to its effectiveness in high dimensional spaces.

**Figure 5:**
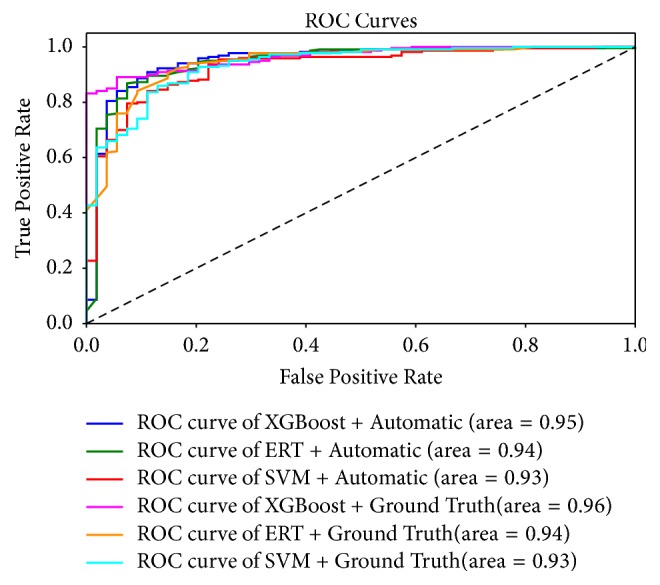
Receiver operating characteristic curve.

In order to assess the robustness and effectiveness of the proposed CAD system, we also carried out our method based on the ground truth segmentation. The classification results are presented in Table 4. The first row in Table 4 shows four performance indices: accuracy, weighted macroprecision, weighted macrorecall, and weighted macro-*F*1 score. It is worth noting that “weighted” means that it takes class imbalance into account and can result in an *F*-score that is not between precision and recall. The values in bold show the best classification rank. The results show that the classification performance is better when using XGBoost, as expected. We also use the receiver operation characteristic (ROC) curve as another method to evaluate the overall performance (Figure 5). For XGBoost, the area under the ROC curve based on automated segmentation is 0.95, while that based on ground truth segmentation is 0.96. From the results above, it can be seen that classification results based on different segmentations have no significant differences.

We further investigate the selected feature subset based on ground truth segmentation, as shown in Table 5. A detailed description of these features can be found in Pyradiomics documentation website (http://pyradiomics.readthedocs.io). As we can see, 3 features from the T1 image and 14 features from the T1c image and 6 features from the T2 image and 2 features from the FLAIR image were selected. Consistent with the selected features based on automatic segmentation, the features of T1c image best reflect the difference between HGG and LGG, and none of the shape features were selected. The features with “*∗*” are also selected in the radiomic signature based on automatic segmentation. There are 12 such features in total, including 1 feature from the T1 image, 7 features from the T1c image, and 4 features from the FLAIR image. This demonstrates that CAD system based on automatic segmentation achieved consistent results with the system based on ground truth segmentation. This is probably because most of the features are based on spatial averaging and highly accurate segmentation results may not be necessary.

## 4. Conclusion

This paper presents an automated scheme using multiparametric MRI scans for differentiating the malignancy of gliomas. A deep learning-based method, called DeepMedic, is used to automatically detect tumor regions. A wide range of radiomic features is extracted from segmentation regions. Using SVM-RFE for feature selection, we construct a 20-feature radiomic signature and train a XGBoost classifier. The results demonstrate that XGBoost-based classification is an objective and promising approach for evaluating the malignancy of gliomas.

Radiomics has been greatly developed in recent years, and its goal is to extract quantitative features as a clinical decision support tool. However, most of these studies are based on manually segmented ROIs, which limit the development of radiomics. Recently, CNN-based approaches, such as DeepMedic, U-net [29], and V-net [30], have achieved great success in the field of medical image segmentation. All of these motivate us to develop a fully automatic method of combining automated brain tumor segmentation with radiomics to develop a fully automated CAD system.

The features that make up the signature contain all MRI modalities. This means all MRI modalities play important roles in our classification task. However, only first-order and texture features are contained in the signature, probably because the shape features of the whole tumor have poor discrimination ability of MR images. In the future work, we will explore the discrimination ability of brain tumor subregions, such as necrosis, edema, active-enhanced tumor, and nonenhanced tumor.

Our work demonstrates that the automatic segmentation of brain tumors can be integrated into computer-aided diagnosis. The classification results based on automated segmentation and ground truth segmentation have no significant differences. This is probably because most of the features are based on spatial averaging; highly accurate segmentation results may not be necessary. Our work provides an alternative idea that we can use automatic segmentation for radiomic features extraction. This will reduce the difficulty of radiomics research since manual segmentation is a time-consuming task.

## Figures and Tables

**Table 1:** Texture features.

Texture feature groups	Abbreviation
Gray level cooccurrence matrix	GLCM
Gray level size zone matrix	GLSZM
Gray level run length matrix	GLRLM
Neighbouring gray tone difference matrix	NGTDM
Gray level dependence matrix	GLDM

**Table 2:** Network architectures.

Structure name	Value
Input channels	T1, T1c, T2, FLAIR
Output classes	Tumor, normal
Pathways	2
FMs/layer	30, 30, 40, 40, 40, 40, 50, 50
FMs/Hidd	150, 150
Seg. norm	25*∗*25*∗*25
Seg. low	19*∗*19*∗*19
Batch size	10

**Table 3:** The feature subset based on automated segmentation.

Selected feature	Group	Modality
Cluster Shade	GLCM	T1
90 Percentile	First-order	T1c
Energy	First-order	T1c
Mean Absolute Deviation	First-order	T1c
Minimum	First-order	T1c
Root Mean Squared	First-order	T1c
Total Energy	First-order	T1c
Small Dependence Emphasis (SDE)	GLDM	T1c
Small Dependence Low Gray Level Emphasis (SDLGLE)	GLDM	T1c
High Gray Level Run Emphasis (HGLRE)	GLRLM	T1c
Low Gray Level Run Emphasis (LGLRE)	GLRLM	T1c
Gray Level Variance (GLV)	GLSZM	T1c
Large Area Emphasis (LAE)	GLSZM	T1c
Maximum	First-order	T2
Correlation	GLCM	T2
Informal Measure of Correlation 1 (Imc1)	GLCM	T2
Informal Measure of Correlation 2 (Imc2)	GLCM	T2
Maximum	First-order	FLAIR
Informal Measure of Correlation 1 (Imc1)	GLCM	FLAIR
Large Area Emphasis (LAE)	GLSZM	FLAIR

**Table 4:** Average performance via 5-fold cross-validation.

	Accuracy	Precision	Recall	*F*1 score
XGBoost + automatic	91.27%	91.27%	91.27%	90.64%
XGBoost + Ground truth	91.25%	91.63%	91.25%	91.06%
ERT + automatic	90.98%	90.94%	90.89%	90.21%
ERT + Ground truth	90.52%	90.50%	90.52%	89.67%
SVM + automatic	90.16%	90.12%	90.16%	89.24%
SVM + Ground truth	90.16%	90.20%	90.16%	89.12%

**Table 5:** The feature subset based on ground truth segmentation.

Selected feature	Group	Modality
90 Percentile	First-order	T1
Cluster Shade^*∗*^	GLCM	T1
Maximum Probability (MP)	GLCM	T1
90 Percentile^*∗*^	First-order	T1c
Kurtosis	First-order	T1c
Mean	First-order	T1c
Mean Absolute Deviation^*∗*^	First-order	T1c
Root Mean Squared^*∗*^	First-order	T1c
Skewness	First-order	T1c
Dependence Nonuniformity (DN)	GLDM	T1c
Small Dependence Emphasis (SDE)^*∗*^	GLDM	T1c
Small Dependence Low Gray Level Emphasis (SDLGLE)^*∗*^	GLDM	T1c
High Gray Level Run Emphasis (HGLRE)^*∗*^	GLRLM	T1c
Low Gray Level Run Emphasis (LGLRE)^*∗*^	GLRLM	T1c
Run Length Nonuniformity (RLN)	GLRLM	T1c
Large Area High Gray Level Emphasis (LAHGLE)	GLSZM	T1c
Small Area High Gray Level Emphasis (SAHGLE)	GLSZM	T1c
Maximum^*∗*^	First-order	T2
Correlation^*∗*^	GLCM	T2
Informal Measure of Correlation 1 (Imc1)^*∗*^	GLCM	T2
Informal Measure of Correlation 2 (Imc2)^*∗*^	GLCM	T2
Dependence Nonuniformity Normalized (DNN)	GLDM	T2
Low Gray Level Zone Emphasis (LGLZE)	GLSZM	T2
Cluster Shade	GLCM	FLAIR
Large Area High Gray Level Emphasis (LAHGLE)	GLSZM	FLAIR
